# Complete Genome Sequence of Enterococcus faecalis Strain SGAir0397, Isolated from a Tropical Air Sample Collected in Singapore

**DOI:** 10.1128/MRA.00593-19

**Published:** 2019-08-15

**Authors:** Rikky W. Purbojati, Daniela I. Drautz-Moses, Akira Uchida, Anthony Wong, Megan E. Clare, Kavita K. Kushwaha, Alexander Putra, Balakrishnan N. V. Premkrishnan, Cassie E. Heinle, Ngu War Aung, Vineeth Kodengil Vettath, Ana Carolina M. Junqueira, Stephan C. Schuster

**Affiliations:** a Singapore Centre for Environmental Life Sciences Engineering, Nanyang Technological University, Singapore; b Departamento de Genética, Instituto de Biologia, Universidade Federal do Rio de Janeiro, Rio de Janeiro, Brazil; University of Maryland School of Medicine

## Abstract

Enterococcus faecalis strain SGAir0397 was isolated from a tropical air sample collected in Singapore. Its genome was assembled using single-molecule real-time sequencing data and comprises one circular chromosome with a length of 2.69 Mbp. The genome contains 2,595 protein-coding genes, 59 tRNAs, and 12 rRNAs.

## ANNOUNCEMENT

Enterococcus faecalis, a Gram-positive, coccus-shaped, facultative anaerobe, belongs to the phylum *Firmicutes* and is commonly found in the gastrointestinal tract of humans (1) and animals (2). It is typically found as a commensal in the human gut but has the potential to cause nosocomial infections (3) as a response to environmental changes, such as antibiotic-mediated alteration of the intestinal microbiome (4) or migration to the bloodstream or other sites in the human body (5).

One common mechanism of dispersal is through the release of human and animal fecal matter from the gastrointestinal tract (6). Enterococcus faecalis has been identified and isolated from exogenous environments such as plants and soils (7), marine water and sediment (8), freshwater (9, 10), and sewage (11). Due to its close association with the gastrointestinal tract, traces of this bacterium in surface water are commonly used as an indicator of human/animal fecal contamination (10, 12).

The strain SGAir0397 was isolated from an air sample collected in Singapore (global positioning system [GPS] location 1.344°N, 103.68°E) using an Andersen single-stage impactor (SKC, USA). The collected air was impacted onto Marine agar (Becton, Dickinson, USA), and further isolation was carried out by cultivation on tryptic soy agar (Becton, Dickinson) at 30°C until axenic growing colonies were obtained. A single colony was inoculated in lysogeny broth (Becton, Dickinson) and grown overnight for genomic DNA extraction. Genomic DNA was purified using the Wizard genomic DNA purification kit (Promega, USA), according to the manufacturer’s protocol. Library preparation was performed with the SMRTbell template prep kit 1.0 (Pacific Biosciences, USA), followed by single-molecule real-time (SMRT) sequencing on the Pacific Biosciences RS II platform.

A total of 80,144 subreads were used for *de novo* assembly with Hierarchical Genome Assembly Process (HGAP) version 3 (13) and polished using Quiver (13) within the PacBio SMRT Analysis 2.3.0 assembly protocol. The polished assembly was then circularized and reoriented with Circlator 1.1.4 (14), resulting in one circular chromosome contig with a length of 2,696,714 bp (217-fold coverage). The chromosomal contig showed a mean G+C content of 37.8%.

Taxonomic identification was performed using the full-length 16S rRNA gene sequence, predicted with Barrnap 0.7 (15), and by determining the average nucleotide identity (ANI) of the genome. The BLASTn (16) result showed 100% sequence identity to Enterococcus faecalis strain CVM N48037F. Meanwhile, ANI analysis (17) resulted in a closest similarity of 98.9% to the Enterococcus faecalis ATCC 19433 genome.

The genome was annotated with the RAST*tk* (18) Web server. A total of 2,595 coding sequences (CDS), 12 rRNA operons (5S, 16S, and 23S rRNAs), and 59 tRNAs were predicted. A CRISPR array with the repeat element GTTTTGGTACCATTCTAAACAACATGACTCTAAAAC spans a total of 432 bp in the chromosome. antiSMASH 5.0.0rc1 analysis (19) resulted in the prediction of a secondary metabolite biosynthesis prediction gene cluster of bacteriocin. Meanwhile, antibiotic resistance gene analysis with ResFinder 2.1 (20) predicted a gene associated with resistance to macrolide, lincosamide, and streptogramin B antibiotics. Default parameters were used for all software programs, unless otherwise specified.

The isolation of this Enterococcus faecalis strain SGAir0397 from the air may provide additional insight into the mode of dissemination and transfer between reservoirs, which could be clinically relevant in the context of hospital-acquired enterococcal infections.

### Data availability.

The complete genome sequence of Enterococcus faecalis strain SGAir0397 has been deposited in the NCBI under accession number CP039434, and the raw sequencing data have been deposited in the NCBI SRA under accession number SRR9043821.
